# Improving Fire Retardancy of Beech Wood by Graphene

**DOI:** 10.3390/polym12020303

**Published:** 2020-02-03

**Authors:** Ayoub Esmailpour, Roya Majidi, Hamid R. Taghiyari, Mehdi Ganjkhani, Seyed Majid Mohseni Armaki, Antonios N. Papadopoulos

**Affiliations:** 1 Department of Physics, Faculty of Sciences, Shahid Rajaee Teacher Training University, Tehran 22970021, Iran; esmailpour@sru.ac.ir (A.E.); royamajidi@gmail.com (R.M.); 2 Wood Science and Technology Department, Faculty of Materials Engineering & New Technologies, Shahid Rajaee Teacher Training University, Tehran 22970021, Iran; mahdi.ganjkhani1@gmail.com; 3 Faculty of Physics, Shahid Beheshti University, Evin, Tehran 22970021, Iran; majid.mohseni19@yahoo.com; 4 Laboratory of Wood Chemistry and Technology, Department of Forestry and Natural Environment, International Hellenic University, GR-661 00 Drama, Greece

**Keywords:** fire retardants, fire retardancy, graphene, nano-materials, wollastonite

## Abstract

The aim of this paper was to improve the fire retardancy of beech wood by graphene. Six fire properties, namely time to onset of ignition, time to onset of glowing, back-darkening time, back-holing time, burnt area and weight loss were measured using a newly developed apparatus with piloted ignition. A set of specimens was treated with nano-wollastonite (NW) for comparison with the results of graphene-treated specimens. Graphene and NW were mixed in a water-based paint and brushed on the front and back surface of specimens. Results demonstrated significant improving effects of graphene on times to onset of ignition and glowing. Moreover, graphene drastically decreased the burnt area. Comparison between graphene- and NW-treated specimens demonstrated the superiority of graphene in all six fire properties measured here. Fire retardancy impact of graphene was attributed to its very low reaction ability with oxygen, as well as its high and low thermal conductivity in in-plane and cross-section directions, respectively. The improved fire-retardancy properties by the addition of graphene in paint implied its effectiveness in hindering the spread of fire in buildings and structures, providing a longer timespan to extinguish a fire, and ultimately reducing the loss of life and property. Based on the improvements in fire properties achieved in graphene-treated specimens, it was concluded that graphene has a great potential to be used as a fire retardant in solid wood species.

## 1. Introduction

Wood is a versatile material with a myriad of applications and therefore, its plantation and harvesting are vastly studied all over the world [1,2]. The idea of protecting solid wood, and wood and cellulose-based materials against different physical and chemical damages, and against the attacks of living micro-organisms, and fire as well, is as old as human civilizations [3,4,5,6,7]. Over time, numerous methods and a variety of materials have been examined and developed. Some methods changed the pathway of pyrolysis in wood cell-wall polymers [8]. This is considered one of the easiest and inexpensive ways for wood. In another method, the surface of the wood is improved, acting as an isolating layer. Intumescent coatings are also categorized in the surface protection method. Alteration in the thermal properties through changing its density, specific and heat thermal conductivity is another one that can also be used to improve fire retardancy in wood and wood-based composites. Other techniques involve decreasing wood ignitability by diluting pyrolysis gases or inhibiting the chain reactions of burning. Though several practical and effective methods and techniques have so far been developed, research for more effective and non-toxic materials to improve fire retardancy is still in progress [9,10,11,12,13,14].

Improving the effects of nanotechnology on different materials has been vastly and intensively elaborated [15,16,17,18,19,20,21]. Wood-based materials and composites are no exception [22,23,24]. Different nano-metals and nano-minerals were utilized to improve heat-transfer property in solid wood species and wood composite mats; they were also used to improve biological resistance against different deteriorating fungi to decrease hot-press time as a costly bottle-neck in nearly all wood-composite manufacturing factories and to increase thermal conductivity in solid wood and composite mats [3,15,18,25,26,27,28,29,30,31,32,33].

In this connection, graphene is a one-atom-thick planar sheet of a hexagonally arranged carbon atoms and therefore, it is considered a nano-material. These sheets are densely packed in a honeycomb crystal lattice. Graphene has got appreciable attention over the last two decades due to its special structure and exceptional properties [34,35]. These properties have made graphene ideal to be used in electronics, sensors, energy-saving devices, different composites, and emerging modern materials, and many other new applications to be investigated in the future. However, the authors came across little or no research projects studying the outcome of graphene as a fire retardant in wood. Therefore, the present research project was carried out to primarily find out if graphene may improve fire retardancy in beech, as an important industrial solid wood species. However, a parallel study was conducted with nano-wollastonite (NW), as a nano-material that has been reported to improve fire retardancy in both solid wood species and wood-composite panels [36,37,38], for comparison purposes. As the surface of wooden bodies and parts in buildings and structures are painted, graphene and NW were mixed with a popular water-based paint as a carrier of the nano-materials. Separate sets of specimens without any paint (plain wood), and with paint that contained no nano-materials, were prepared and tested for comparison purposes with the graphene- and NW-treated specimens.

## 2. Materials and Methods

### 2.1. Specimen Preparation

For the present research project, beech boards (*Fagus orientalis*) were purchased from the Khavaran Wood Bazar (Tehran, Iran). The density of beech specimens was measured to be 0.62 g·cm^−3^. Boards were kept in the wood workshop of Shahid Rajaee Teacher Training University (Tehran, Iran) for four months before cutting (35–40 °C; relative humidity 26%–30%). They were then cut to size, 220 mm in length, 140 mm in width (plain sawn), and 5 mm in thickness. Specimens were free from any fungal or insect attack, checks or cracks, and knots. Twenty specimens were selected and divided into four treatments; for each treatment, five replicate specimens were prepared. Treatments included: control (without any paint and nano-material), painted, NW+painted (nano-wollastonite was mixed in paint and applied on the surface of specimens), and NG+painted (graphene was mixed with paint and applied on the surface of specimens. Acrylic paints are popular in wood products [39] and therefore, a water-based acrylic-latex paint was used in this experiment (code number ALCO-6510), purchased from Alvan Paint and Resin Production Co. (Tehran, Iran). The solid content of the paint was 37% ± 1%. Two coats were brushed on each specimen to achieve a 190–200 μm dry paint film on the front and back surfaces of specimens. A 24-h time was given between the two coats to let the first coat being dried out. Once the two coats were applied, all specimens were kept in a conditioning chamber (25 ± 1 °C, and 40% ± 2% relative humidity) for two weeks. For the nanomaterial-treated specimens, 15% of nano-material was mixed with the paint, based on the wet weight of the paint, before being brushed on specimens. NW gel was produced by Vard Manufacturing Company of Mineral and Industrial Products (Iran). At least 70% of NW particles ranged from 30 to 110 nm. The formulation of NW gel has been reported by Taghiyari et al. [29,30,31]. Nanomaterials were mixed with the paint for 20 min, using a magnetic stirrer. While mixing the nanomaterials, solid content of the final paint was kept constant by the addition of a calculated amount of distilled water. The moisture content of specimens at the time of fire tests was 8% ± 0.5%.

### 2.2. Graphene Production Technique

Electrochemical exfoliation of graphite was performed in a system with a cathode electrode of platinum (Pt 0.5 × 10 cm^2^) and the anode electrode of graphite foil (2 × 10 cm^2^). Two electrodes were placed at a distance of 2.7 cm from each other. NiCl_2_.6H_2_O powder (98.0% Merck) was dissolved in water (concentration of 0.05 M, and pH 6.5−7.0) to prepare electrolyte. To provide expansion, exfoliation of graphite, and deposition of Ni, a voltage of 10 V was applied. The Pt electrode was washed every 20 min with HCl and water to avoid the accumulation of the product on the cathode. Finally, the products were collected using vacuum filtration, and then they were washed with water. The end product was dispersible for sonication in water. In the electrochemical exfoliation mechanism, hydroxyl ions (OH) were first produced in the cathode region by applying the voltage between the electrodes, and then the ions accelerate towards the anode and hit the graphite surface. The collision of OH ions with graphite and oxidation at the edge sides and grain boundaries lead to expansion of the graphite layers, penetration of Cl ions through the graphite layers, and reduction of Cl ions to finally produce Cl gas. An excessive force exerted to graphite layers upon Cl gas caused the separation of the graphite layers [40,41]. Then, graphene sheets distributed in the solution trapped Ni^2+^ ions and proceeded towards the negative electrode under an electric field. Finally, black composite on the Pt electrode was created. Generation of hydroxyl at cathode via ionization of water is as follows [40]:

2H_2_O + 2e→H_2_ + 2OH.

The OH generation together with other electrons and ions lead to formation of Ni and Ni(OH)_2_ on graphene sheets. Finally, Ni and Ni(OH)_2_ as crystalline layers were deposited on graphene flakes (G-flakes). The final product in form of powders was pressed (under a pressure of 5 MPa for 30 min) into pellets. The average dimensions were 6.5 mm (radius) and 1 mm (thickness), approximately.

The characterization of Ni-graphene composite was done by X-ray diffraction (XRD, Cu Kα λ = 0.154 nm) radiation, X-ray photoelectron spectroscopy (XPS, ESCA/AES, CHA, Specs model EA10 plus), tunneling electron microscopy (TEM-Philips model CM120), and dynamic light scattering (DLS, Malvern Instruments Ltd., Worcestershire). TEM and XRD patterns of Ni-graphene represented that the nano-crystals (Ni, Ni(OH)_2_, Ni-oxides) were randomly distributed on graphene sheets. The size of Ni and Ni(OH)_2_ nanocrystals obtained by TEM and XRD patterns were found to be 30–40 nm. The brightness of graphene sheets appearing in TEM images showed a small thickness (1.2 μm) of graphene sheets [42].

### 2.3. Fire Test Apparatus

**Figure 1:**
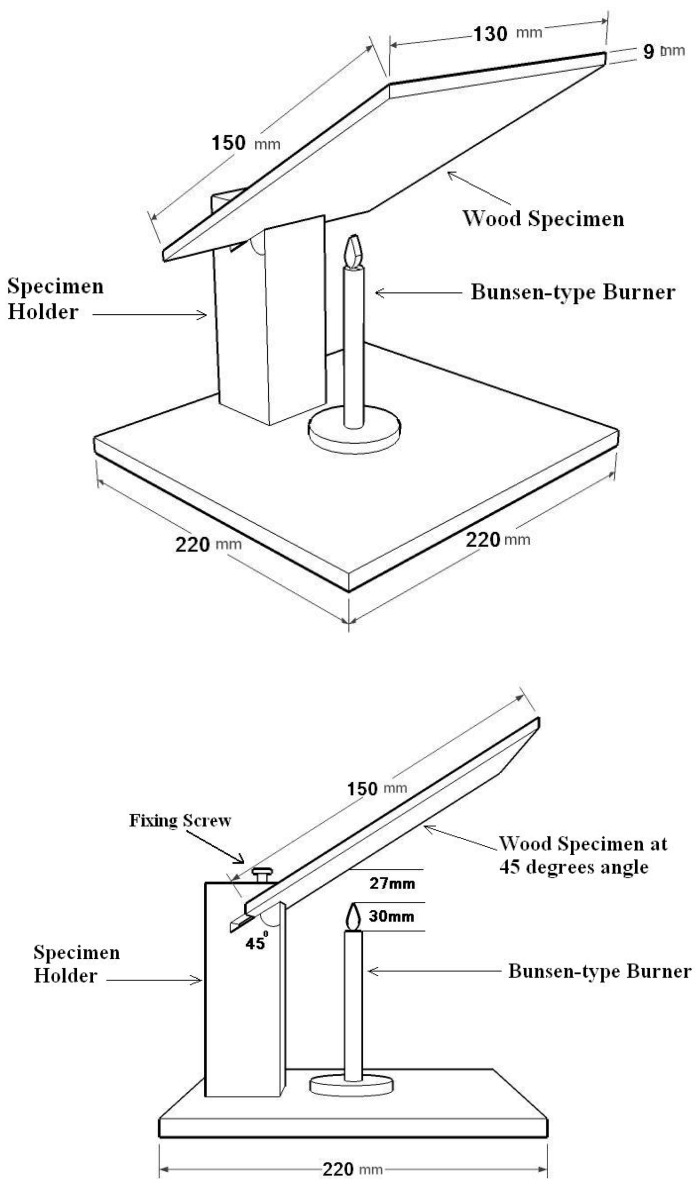
Schematic picture of fixed fire testing apparatus (Iranian Patent No. 67232; approved by Iranian Research Organization for Science and Technology under license No. 3407; USPTO Pub. No.: US 2019/0212283 A1) [38,43].

Fixed Fire Test Apparatus (FFTA) was designed and built, using piloted ignition, as depicted in Figure 1 [39,43]. Natural gas was used as the fuel; it mainly comprised of methane CH_4_ (90–98%). The producer reported that other hydrocarbons accompanied methane (C_2_H_6_: 1–8%; C_3_H_8_: 2%; H_4_H_10_+C_5_H_12_: less than 1%; and also N_2_ + H_2_S + H_2_O: less than 1.5%). The gas flew steadily at the rate of 0.097 lt/s through a Bunsen type burner hold vertically and the specimen is mounted at a 45° angle to it. The internal diameter of the burner was 11 mm. The Bunsen-type burner provides a fairly mild and localized fire exposure to the testing specimens. The time from the point of burner application that it takes for the specimen to develop a visible flame that is sustained for more than one second will be registered as the “time to onset of ignition”; and the time from the point of burner application that it takes for the specimen to sustain glowing for more than one second will be registered as the “time to onset of glowing”. The higher the amount of time of these two properties, the better from the viewpoint of fire-safety. As the burning continues, the back face of the specimen nearest to the flame of the burner starts blackening and after some more time, a small hole or split appears. These times are also registered as back-blackening and back-holing times. The test is terminated once the back-holing occurs. Then, the burnt area, as well as weight loss, is measured. The whole apparatus is put in a three-wall-compartment in order to protect the burning flame from wind and air movements.

### 2.4. Computational Modeling and Simulation

Density functional theory (DFT) calculations were carried out for graphene and oxygen molecule with OpenMX3.8 package to calculate the adsorption energy of oxygen molecule on graphene [44,45,46]. The generalized gradient approximation (GGA) function with the Perdew-Burke-Ernzerhof (PBE) correction was employed to analyze the exchange-correlation energy functional between graphene and oxygen molecule [47]. The energy cutoff was set 100 Ry. The van der Waals interactions between adsorbed molecules and sheets were described with Grimme′s method [48].

**Figure 2:**
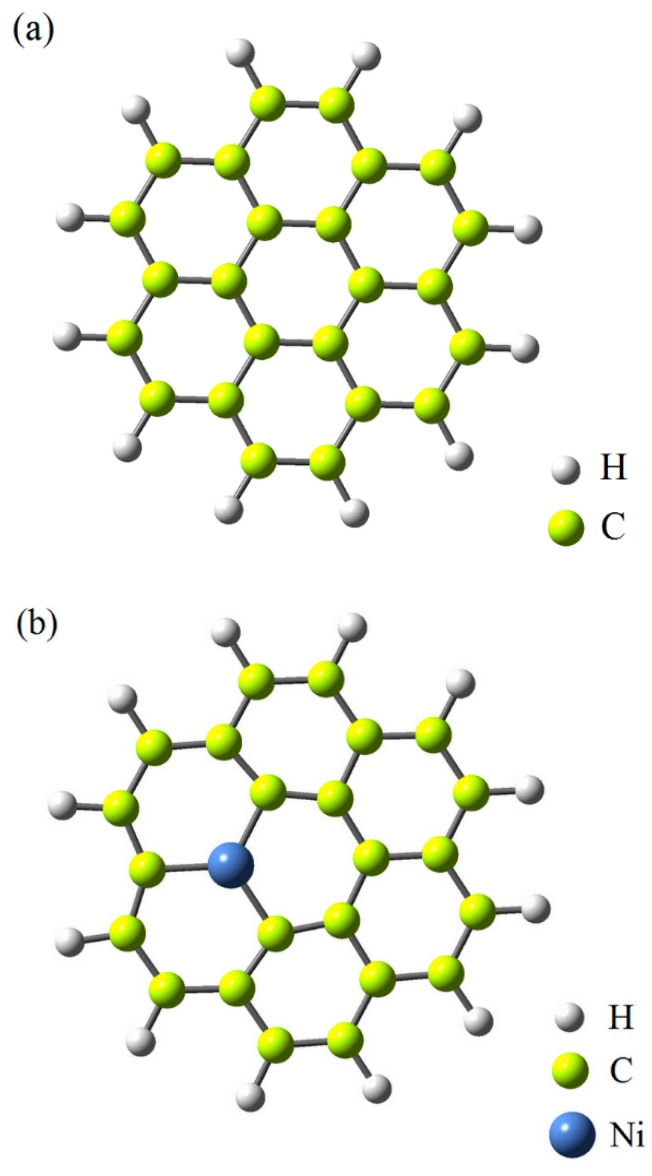
Atomic structure of pure (**a**) and Ni-doped (**b**) graphene flakes.

In the present simulations, one model for pure graphene flake (G-flake) was investigated. The fully optimized atomic structure of pure G-flake is shown in Figure 2a. This G-flake consists of 24 C atoms. The edge C atoms of the G-flake were terminated by H atoms. The second model represents Ni-doped G-flake in which one of C atom in the pure G-flake was replaced by a Ni atom to model Ni-doped G-flake. The optimized structure of G-flake doped with Ni atom is shown in Figure 2b.

The adsorption energy, $E_{Ads}$, was calculated to evaluate stability of structures by the Equation (1).

(1) $$E_{Ads}=E_{G-flake+O_{2}}-(E_{G-flake}+E_{O_{2}}),$$

where $E_{G-flake+O_{2}}$ is the total energy of G-flake with adsorbed oxygen molecule, $E_{O_{2}}$ and $E_{G-flake}$ are the total energies of oxygen molecule and G-flake, respectively.

### 2.5. Statistical Analysis

Statistical analysis was carried out by a SAS software, version 9.2 (Cary, NC, USA). One-way analysis of variance (ANOVA) was performed on the average values to ascertain significant differences at the 95% level of confidence. Hierarchical cluster analysis, including dendrograms and Ward methods (using squared Euclidean distance intervals), was carried out using SPSS/18, version 18 (IBM, Armonk, NY, USA). The scaled indicator on top of cluster analysis shows similarities and differences between treatments; lower scale numbers show more similarities while higher ones show dissimilarities. Contour and surface plots were designed in Minitab software, version 16.2.2 (Minitab Inc., Philadelphia, PA, USA).

## 3. Results and Discussion

**Figure 3:**
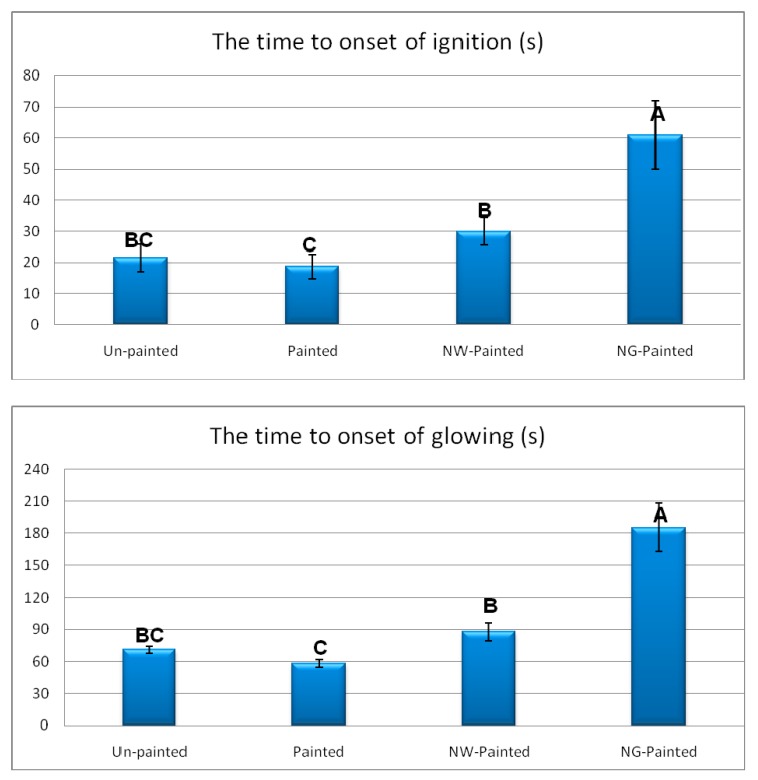
Time to onset of ignition and glowing, in the four treatments of beech specimens (NW = nanowollastonite; NG = nano-graphene) (Letters on each column represent Duncan groupings at 95% level of confidence).

**Figure 4:**
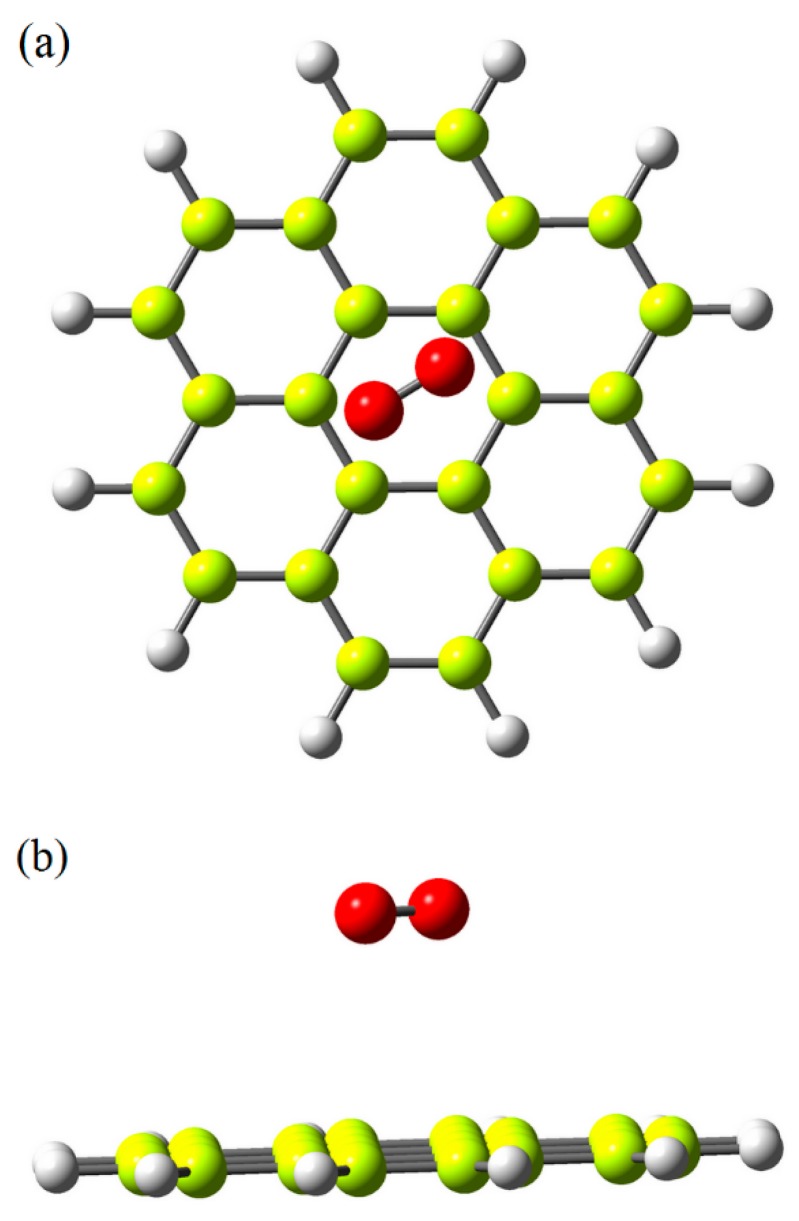
Side (**a**) and top (**b**) views of oxygen molecule adsorbed on pure graphene flake.

**Figure 5:**
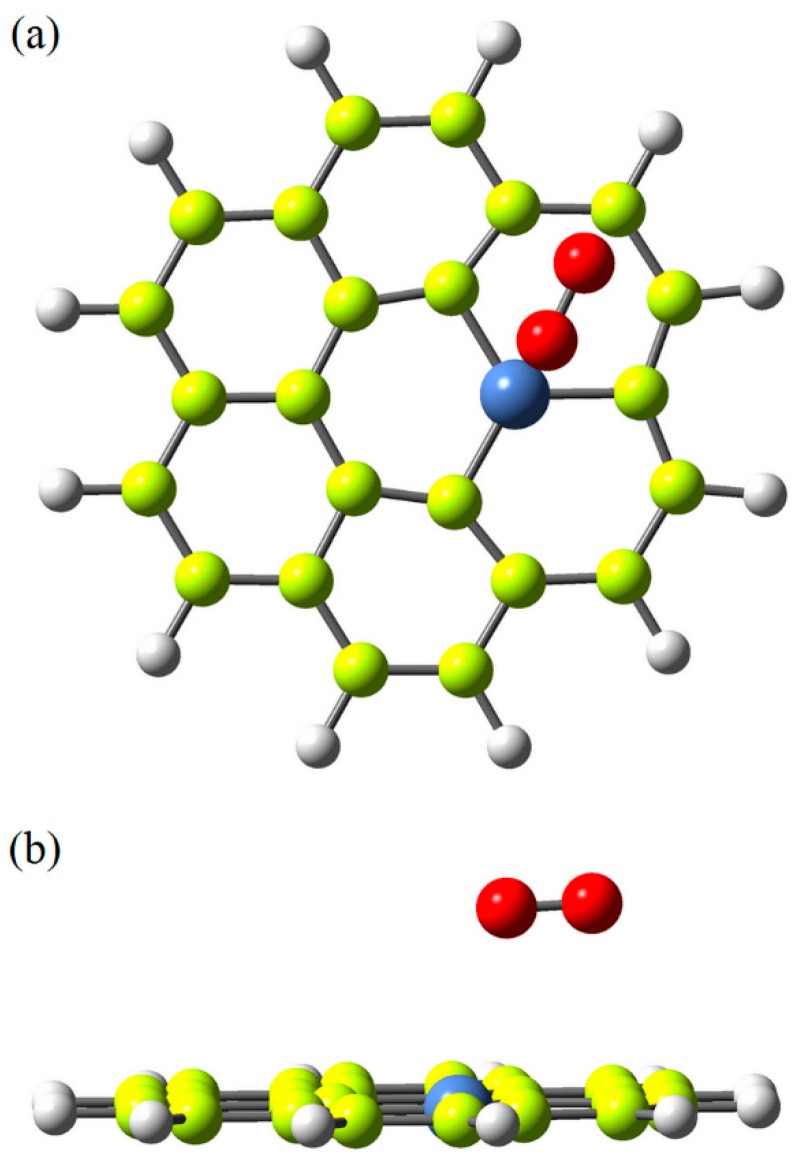
Side (**a**) and top (**b**) views of oxygen molecule adsorbed on Ni-doped graphene flake.

Results demonstrated outstanding improving effects of graphene (NG) on both times to onset of ignition and to the onset of glowing (Figure 3). NG resulted in 184% and 162% increases in times to onset of ignition and glowing, respectively, in comparison to un-painted specimens. The favorable increases are primarily attributed to the very low reaction ability of graphene with oxygen [49]. A weak bond with basically no charge transfer was reported to form between oxygen molecules and graphene sheets or tubes with very low adsorption energy [49,50]. In fact, graphene here acted as an impermeable insulating layer towards penetration of fire into its substrate, significantly delaying in its ignition and glowing. DFT analysis in the present study also revealed that energy of oxygen molecule on Pure and Ni-doped G-flakes were −1.07 and −1.20 eV, respectively (Figure 4 and Figure 5). The shortest distances from O atom in oxygen molecule to C atom in pure and Ni-doped G-flakes were calculated to be 3.40 and 2.37 Å, respectively. These small adsorption energies and large adsorption distances clearly demonstrated that oxygen molecules were weakly physisorbed by pure and Ni-doped G-flake, ultimately illustrating very low reaction ability between them.

The improvement in fire properties by graphene can be elaborated from its thermal conductivity as well. With regard to its two-dimensional structure, the thermal conductivity of graphene can also be studied in two directions. The in-plane thermal conductivity of graphene is reported to be one among materials the highest thermal conductivity, about 2000–4000 W·m^−1^·K^−1^, at room temperature [51]. For comparison purposes, the thermal conductivity of natural diamond is about 2200 W·m^−1^·K^−1^, and that of a purified diamond can be up to 50% higher. However, the thermal conductivity of graphene in cross-section direction (that is, along the nano-size direction of *z*-axis) is as low as only 6 W·m^−1^·K^−1^ [51]. This extreme difference in thermal conductivity of in-plane and cross-section directions played a vital role in improving fire properties in beech specimens. From one perspective, very low thermal conductivity in cross-section direction prevented heat flow from the piloted ignition to pass through and reach the beech wood substrate, delaying the substrate in catching fire, ultimately improving fire properties. The increases in both times to onset of ignition and glowing partially resulted from this low thermal conductivity in cross-section direction. From another perspective, the high thermal conductivity in the in-plane direction prevented accumulation of heat at the point nearest to the piloted fire, eventually postponing its ignition. Similar transfer of heat to the surrounding area of woody specimens by nano-wollastonite was previously reported to improve fire properties as well [36,38]. Overall, the thermal conductivity of graphene in both directions helped its improving fire properties in beech specimens.

Painted specimens showed a little bit of decrease in time values, though the decreases were not statistically significant. The decrease, which is not favorable as far as fire retardancy is concerned, was attributed to the easier ignitability of the chemical ingredients of the paint applied on the surface of specimens. The addition of NW to the paint resulted in an increase in both times to onset of ignition and glowing in comparison to the unpainted specimens, though the increase was not statistically significant in time to onset of glowing. The increase was partially attributed to the mineral and unignitable nature of wollastonite. In fact, NW acted as an insulating layer, too, towards the penetration of the piloted fire from the Bunsen burner, protecting the wood substrate beneath the paint.

**Figure 6:**
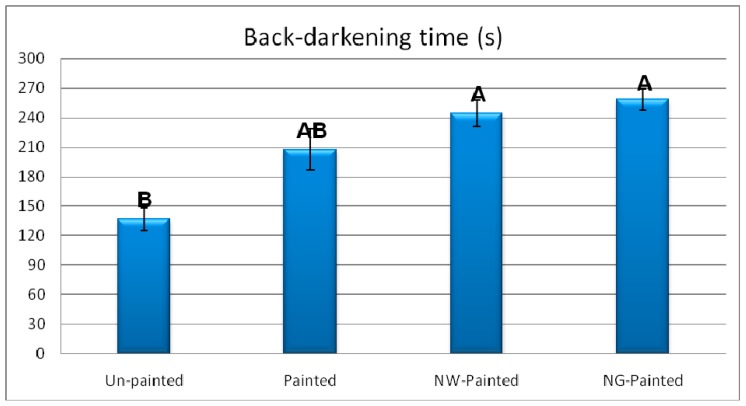

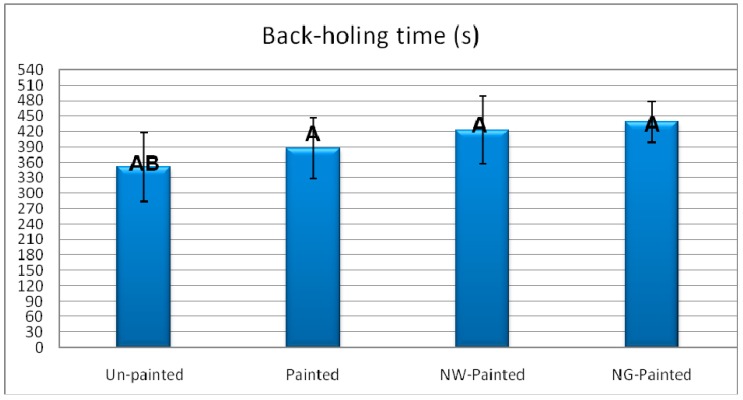
Back-darkening and back-holing times, in the four treatments of beech specimens (NW = nanowollastonite; NG = nano-graphene) (Letters on each column represent Duncan groupings at 95% level of confidence.).

Results of assessing the back of specimens showed that both NG and NW increased the times to back-darkening and to back-holing (Figure 6), showing they are both reliable to hinder the passing of fire through specimens. NG performed a bit more efficiently with regard to these two fire-retarding properties in relation to the backside properties of specimens. This may be attributed to the unignitable nature of graphene, acting an insulating layer towards penetration and passing of flame.

**Figure 7:**
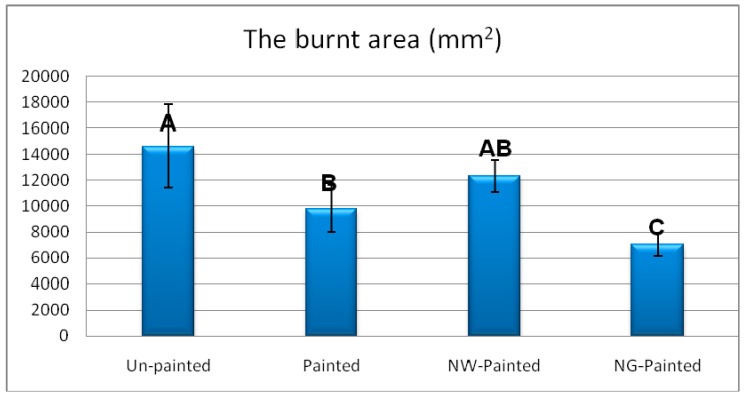

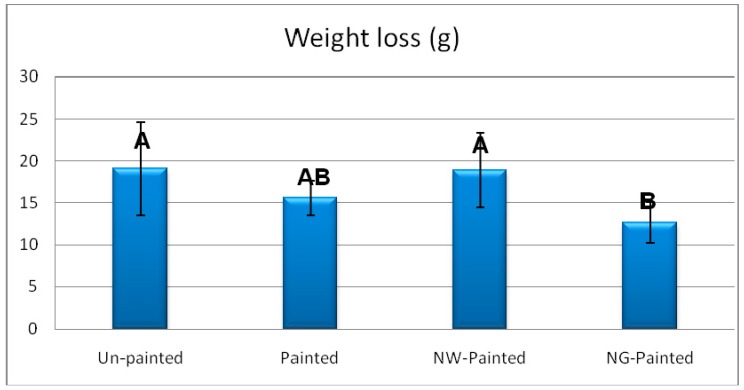
Burnt area and weight loss in the four treatments of beech specimens (NW = nanowollastonite; NG = nano-graphene) (Letters on each column represent Duncan groupings at 95% level of confidence.).

Measurement of the burnt area illustrated that NG was very effective in hindering the spread of fire throughout specimens (Figure 7). The spread of fire in materials made of wood is of vital importance as its decrease would limit the potentiality of other surrounding parts to catch on fire. A decrease in the burnt area would eventually provide fire-fighters with a longer time-span to extinguish the fire. This would ultimately result in a decrease in losses, both to life and property. As to NW, thought it decreased the burnt area in comparison to un-painted specimens, the area was bigger than that of NG. This was attributed to an increased thermal conductivity that caused heat to be more easily transferred to the surrounding areas, ultimately increasing the burnt area. Similar increase in thermal conductivity was previously reported [22,30].

The weight measurement of specimens before and after being exposed to fire showed that the lowest weight losses occurred in specimens treated with NG (Figure 7). The weight loss of NG-painted specimens was 50% lower than that of the control of unpainted specimens. This clearly indicated that NG can significantly decrease the volume of burnt materials.

**Figure 8:**
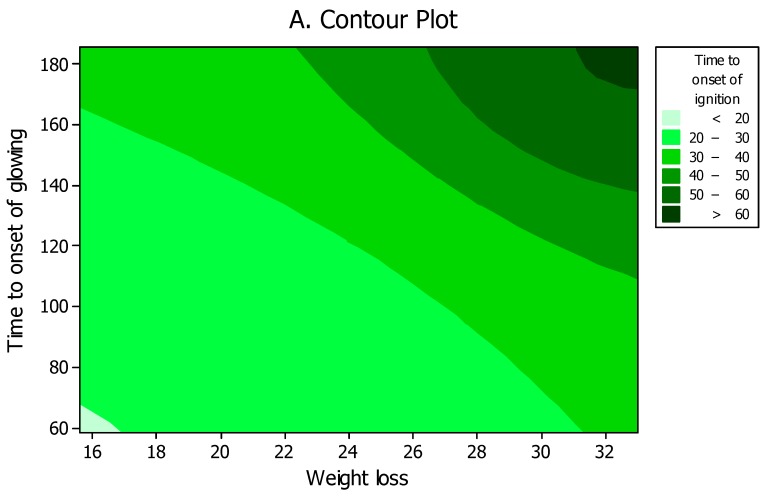

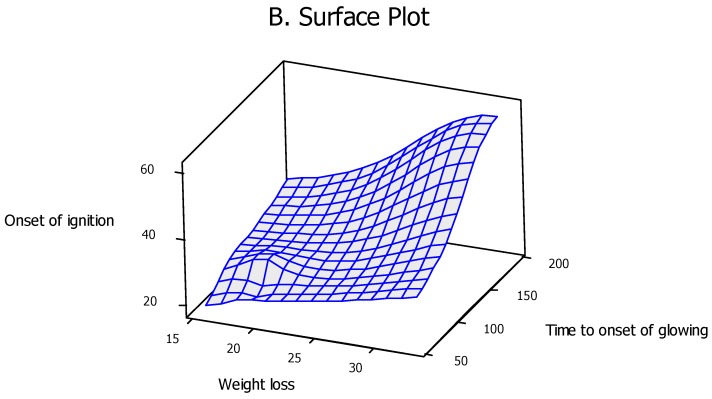
Contour (**A**) and surface plots (**B**) among fire properties of weight loss versus times to onset of glowing and ignition.

A significant direct relationship was calculated between most of the fire properties. The highest R-square (99%) was found between the two times to onset of ignition and glowing. Contour and surface plots also illustrated a direct trend among different properties; for instance, weight loss increased as the two times to onset of glowing and ignition increased (Figure 8A,B).

**Figure 9:**
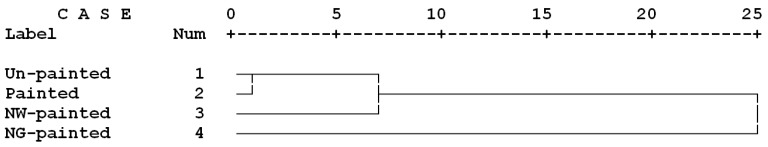
Cluster analysis among the four treatments of beech specimens based on all the fire properties studied in the present project (NW = nano-wollastonite; NG = nano-graphene).

The cluster analysis based on all six fire properties studied in the present project demonstrated the distinct difference of specimens treated with graphene in comparison to the other three treatments (control, painted, and NW-painted) (Figure 9). This indicated the great improving impact of graphene on fire properties in beech specimens. Painted specimens were closely clustered with the control specimens, indicating no significant difference between these two treatments. NW-painted specimens were in-between position of control and NG-painted; this indicated significant improving effects of NW on fire properties, similar to previous studies on solid wood and wood-composite panels [36,38]. However, the difference in clustering with NG-painted specimens showed higher effectiveness of graphene in improving fire properties in comparison to NW. Based on the results discussed above, it was concluded that graphene has great potential in improving fire properties in beech. Easy application of graphene on the surface of materials makes it a good prospect for the industry sector to develop an effective fire-retardant. However, as the paint used in the present project can even be more elaborated to find a better carrier liquid.

## 4. Conclusions

Fire properties of beech specimens were tested by a newly developed apparatus using piloted fire. Specimens were prepared to be surface-treated by graphene to investigate its improving effects on fire properties. Graphene was mixed at 5% with water-based paint and applied on the front and back surfaces of specimens. As a basis for comparison, three separate groups of specimens were prepared, including specimens without any paint (the control group), painted with no nano-material mixed with it, and painted with 5% nano-wollastonite. Results showed significant improving effects of graphene on all six fire properties studied in the present project. Graphene demonstrated outstanding improvement in times to onset of ignition and glowing, as two very important properties that determine the ignitability of materials. Moreover, graphene illustrated a high impact on decreasing the burnt area. The decrease was also important from the viewpoint of the spread of fire to other surrounding parts in an area. Overall, the improved fire properties provide fire-fighters with a larger timespan to extinguish the fire, ultimately saving both life and property. It was concluded that graphene has a great potential to be used as an effective fire-retardant in wood and wood-composite materials for surface protection against fire. Therefore, further studies should be carried out to investigate graphene from other perspectives, including FT-IR analysis.
